# Incidence and associated factors of medication-related osteonecrosis of the jaw in breast cancer patients with bone metastases: a nationwide registry study in Finland

**DOI:** 10.1007/s00784-026-07171-5

**Published:** 2026-09-29

**Authors:** Miika Kujanpää, Ville Vuollo, Antti Tiisanoja, Marja-Liisa Laitala, Jussi Koivunen, George K. Sándor, Saujanya Karki

**Affiliations:** 1 Research Unit of Population Health, University of Oulu, Aapistie 3, POB 5281, Oulu, FI- 90220 Finland; 2 The Wellbeing Services County of North Ostrobothnia, Oulu, Finland; 3 Medical Research Center Oulu, Oulu University Hospital, Oulu, Finland; 4 Department of Oncology and Radiotherapy, Oulu University Hospital, Oulu, Finland; 5 Medical Research Center Oulu, Oulu, Finland; 6 Cancer and Translational Medicine Research Unit, University of Oulu, Oulu, Finland

**Keywords:** Osteonecrosis, MRONJ, Breast cancer, Denosumab, Bisphosphonates

## Abstract

**Objectives:**

This study evaluated the incidence of MRONJ and associated factors among breast cancer patients with bone metastases in Finland.

**Materials and methods:**

This retrospective cohort study included all adult patients with breast cancer and bone metastases who were prescribed antiresorptive therapy between 2013 and 2015. Patients were identified from national health care registers and followed through 2020. The outcome variable was MRONJ (M87.1). Covariates included age, oral diseases, dental procedures, type of antiresorptive (AR) prescribed, comorbidities, and concomitant medications.

**Results:**

A total of 652 patients met the inclusion criteria. The incidence of MRONJ ranged from 0% to 2.2% among bisphosphonate users, compared with 12.6% among denosumab users and 15.3% among patients receiving sequential bisphosphonate and denosumab therapy. Both denosumab monotherapy and sequential bisphosphonate-denosumab therapy were associated with substantially increased MRONJ risk compared with bisphosphonate therapy.

**Conclusions:**

Denosumab use and infection-related invasive dental procedures showed the strongest associations with MRONJ in this study population.

**Clinical relevance:**

MRONJ incidence differed by type of antiresorptive therapy, with higher occurrence among patients using denosumab or bisphoshonates followed by denosumab compared with bisphosphonates. MRONJ was more frequent among patients who underwent infection-related invasive dental procedures.

## Introduction

Breast cancer is the most common cancer worldwide, accounting for 11.7% of all cancer cases [1]. It is the fifth most common cause of cancer-related death (6.9%), following lung, colorectal, liver and stomach cancers [2]. In Finland, a cumulative incidence of breast cancer was 158 per 100,000 in 2022, highest among females over 70 years old [1]. Breast cancer is also the second most common cause of death (13.9%) among females in Finland [3].

Antiresorptive drugs (AR) are commonly used in low doses as an adjuvant therapy with postmenopausal primary breast cancer patients to protect bone health, prevent cancer recurrence, and in higher doses in metastatic disease to reduce bone destruction, alleviate pain, and prevent skeletal-related events. ARs not only increase bone mass and density but may also induce breast cancer cell apoptosis and inhibit cell migration and invasion [4–6].

Antiresorptive medications have been associated with an adverse event called Medication-Related Osteonecrosis of the Jaw (MRONJ). The American Association of Oral and Maxillofacial Surgeons (AAOMS) provides the most widely accepted definition of MRONJ. According to AAOMS, MRONJ involves an exposed bone or bone that can be detected through an intraoral or extraoral fistula in the jaws and has endured for more than eight weeks. This condition typically occurs in patients who have received current or previous treatment with antiresorptive or antiangiogenic agents, and have no history of radiation therapy to the jaw or evident metastatic disease in the jaw [7–10].

MRONJ is common among patients diagnosed with cancer and secondary bone metastasis, or high-dose AR recipients. First studies on the MRONJ risk included only bisphosphonate-treated breast cancer patients, and they reported risks between 0.3% and 1.5% depending on the used bisphosphonate. Recent studies have included both denosumab and bisphosphonate patients, either as a monotherapy or as a sequential use [11]. In denosumab-treated cancer patient studies, the incidence has varied between 1.1% and 5.7% after two-year follow-up and has been as high as 9.8% after four-year follow-up [12, 13]. In studies including both denosumab and bisphosphonates, the risk of MRONJ has varied between 8.8% and 16.3%. Patients treated with denosumab or with both bisphosphonates and then followed by denosumab have consistently been in the highest risk. Although, depending on the study, the highest risk has existed either among sequential users or denosumab-only user [14–16]. In our previous study among a Finnish adult population, over 25% of oncology-dose AR recipients had breast cancer diagnosis. The total incidence of MRONJ differed from 0.32% to 16% depending on the purpose of drug use and dosage [17].

MRONJ has a multifactorial pathophysiology involving cellular, vascular, and microbial processes, alongside antiresorptive drug use. ARs inhibit osteoclast-mediated bone resorption by reducing osteoclast function and promoting apoptosis, leading to suppressed bone turnover and impaired remodeling. Both systemic and local risk factors play a part in the development process. The most recognized systemic risk factors for MRONJ include sequential use of bisphosphonates and denosumab, concomitant medications, especially corticosteroids and antiangiogenic drugs; and systemic conditions such as diabetes, chemotherapy, smoking and older age [18–20]. Notably, most of these systemic risk factors compromise immune function, thereby increasing susceptibility to infections, which are currently considered one of the most crucial contributors to MRONJ pathogenesis [21, 22]. Of the local risk factors, oral infections and traumas caused by extractions, ill-fitting prostheses are the most recognised ones and expose the jawbone to infection, further elevating the risk [12, 19, 23].

Breast cancer patients represent a major group receiving high-dose antiresorptive therapy both in Finland and globally. Alongside prostate cancer patients, they are among those at the highest risk of developing MRONJ. However, to our knowledge, no previous study has been conducted in Finland in line with this area. Therefore, the aim of this study was to evaluate the incidence of MRONJ in breast cancer patients with bone metastasis receiving antiresorptives in Finland. Another aim was to investigate associated oral diseases and related dental procedures as well as concomitant use of certain medications with MRONJ in breast cancer patients with bone metastasis.

## Methods

### Study design and participants

This retrospective study utilized the data acquired from the national registries of Finland (Finnish Institute for Health and Welfare (THL) database and the Social Insurance Institution of Finland (Kela). The THL database consists of two separate registries Hilmo and AvoHilmo. The Hilmo registry concentrates on specialized health care whereas the AvoHilmo covers primary healthcare registries in means of all the healthcare data to be utilized in decision-making, planning and research.

**Fig. 1:**
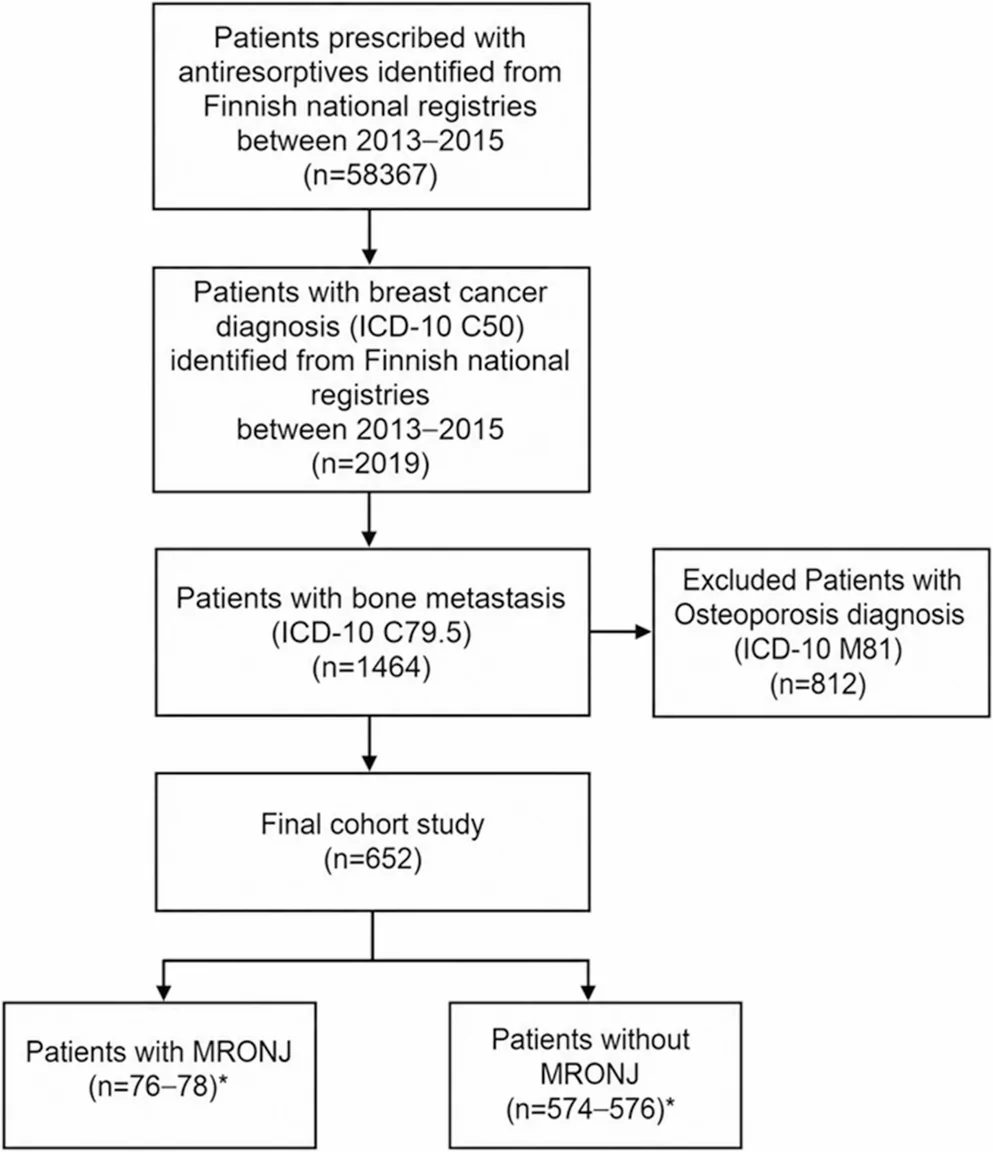
Flowchart of patient selection and cohort formation among Finnish breast cancer patients with bone metastases receiving antiresorptive therapy during 2013–2015. *Due to secondary Use Act, frequency must be anonymous [24]

All adult patients (aged ≥ 18 years old) diagnosed with secondary malignant neoplasm of breast with bone metastasis (ICD-10 diagnosis code C50 and C79.5) and receiving at least one antiresorptive drug (AR) during 2013–2015 were included in this study. The Anatomical Therapeutic Chemical (ATC) codes MB05BA01-07 (bisphosphonates), MB05BB01-09 (bisphosphonate combinations), and MB05BX04 (denosumab) were used as a definition for AR. Patients with concomitant osteoporosis diagnosis (M81) were excluded. Patients meeting the inclusion criteria were included and followed up until 2020 (Fig. 1).

### Ethical issues

This study utilizes secondary data from national registries, therefore separate ethical approval was not necessary. Since the study is retrospective, Social Insurance Institution of Finland (Kela) and Finnish Institute for Health and Welfare (THL) waived the requirement for informed consent. However, in compliance with the legislation on the secondary use of social and health data, a separate permit was obtained from the Finnish Social and Health Data Permit Authority (Findata, permission number THL/3317/14.02.00/2021). Anonymous data was requested and provided by Findata. The Act on the Secondary Use of Health and Social Data requires Findata to provide specific regulations on secure operating environments, data descriptions, data permit applications, and data utilization plans. Relevant guidelines and regulations were followed throughout the study methods. In accordance with Finnish data protection legislation on the secondary use of health and social data, exact frequencies below five cannot be reported in published results to prevent indirect identification of individuals. Therefore, some frequencies and total counts are presented as ranges or approximate values. All statistical analyses, including incidence calculations and regression models, were nevertheless performed using the exact underlying patient counts available in the pseudonymized dataset [24].

### Outcome variable

The outcome variable was the diagnosis of MRONJ among breast cancer patients, who had likely received oncology-dose antiresorptive therapy during their cancer treatment. MRONJ diagnosis was based on the International Classification of Diseases 10th Revision ICD-10 code M87.1. In Finland, M87.1 is commonly recognized as an ICD-10 code for the diagnosis of medication-related osteonecrosis of the jaw^14^.

### Covariates

Data on age, oral diseases, oral procedures, type of AR prescribed, comorbidities and concomitant medication were used as covariates. Covariates were selected based on their clinical relevance and previously reported associations with MRONJ. Variables with a p-value < 0.10 in the univariable analyses were considered for inclusion in the adjusted model. Given the limited number of MRONJ events, the final model was restricted to the most clinically relevant covariates to reduce the risk of overfitting. These data were obtained from the National Drug Purchases Register, AvoHilmo and Hilmo databases.

Oral diseases were identified using ICD-10 codes: dental caries *(K02.1)*, pulpitis and periapical infections *(K04)* and periodontitis *(K05.2-K05.6)*. Comorbidities were defined as hypothyroidism *(E03.9)*, diabetes *(E08-E13)*, hypertension (*I10)*, Asthma *(J45)*, and inflammatory polyarthropathies *(M05-M14).* Concomitants medications were classified using ATC codes: corticosteroids *(H02)*, endocrine therapy *(L02)*, immunostimulants *(L03)*, lipid-modifying agents *(C10)*, and proton pump inhibitors *(A02BC)* and vascular endothelial growth factors (VEGFs) *(L01)* [25].

### Statistical methods

All statistical analyses were performed using IBM-SPSS Statistics (version 26, SPSS, Inc., Chicago, Ill., USA) and R software version 4.2.2 (R Core Team 2022). For statistical analyses covariates were categorized as age (*≤ 64 years and ≥ 65 years*), oral diseases *(dental caries*,* pulpitis and periapical infection*,* periodontitis)*, oral procedures *(root canal treatment*,* periodontal treatment*,* tooth extraction)*, type of AR prescribed *(bisphoshonates*,* denosumab*,* and sequential use)*, comorbities *(hypothyroidism*,* diabetes*,* hypertension*,* asthma)* and concomitant medication.

Proportions were computed, and group differences were assessed using the chi-square test. Associations between the outcome variable and covariates were examined with Cox regression analysis, yielding hazard ratios (HRs) and 95% confidence intervals (CIs). Fisher’s exact test was used when expected cell counts were below five. The proportional hazards assumption was assessed visually and was not violated. The adjusted model included variables with a p-value < 0.10 in the univariable analyses.

## Results

The cohort consisted of *n* = 652 patients. The mean age was 72.3 years and over 99% of them were female. Of the AR drugs, 14.8–15.6% (*n* = 85–90) had received bisphosphonates, 74.4–74.1% (*n* = 427) denosumab, and 23.8–23.9% (*n* = 137) both therapies. MRONJ developed in approximately 12% (*n* = 76–78) of patients (Tables 1 and 2) and the vast majority of the them were either sequential or denosumab users (Table 3).

**Table 1:** Characteristics of patients with and without medication-related osteonecrosis of the jaw (MRONJ) in Finnish women with breast cancer

	Patients without MRONJ *n* (%)	Patients with MRONJ *n* (%)	*P* value
Age group			
≤ 64 years	146–148 (24.0%–26.6%)	25–27 (33.0%–34.0%)	0.132
≥ 65 years	427–429 (74.4%–76.0%)	50–52 (66.0%–67.0%)	
Oral diseases			
Dental caries	70 (12.2%)	22 (28.6%)	< 0.001
Pulpitis & periapical infection	62 (10.8%)	28 (36.4%)	< 0.001
Periodontitis	< 3 (< 0.4%) *	< 3 (< 3.0%)	0.013
Dental procedures			
Root canal treatment	< 3 (< 0.4%) *	5 (6.5%)	< 0.001
Periodontal treatment	14 (2.4%)	5 (6.5%)	0.061
Tooth extraction	76 (13.2%)	40 (51.9%)	< 0.001
Antiresorptive drug			
Bisphosphonates	85–87 (14.0%–16.0%)	< 3 (< 3.0%) *	0.002
Denosumab	373 (64.9%)	54 (70.1%)	
Both	116 (20.2%)	21 (27.3%)	
Comorbidities			
Hypothyroidism	58 (10.1%)	7 (1.1%)	1.000
Diabetes	84 (14.6%)	13 (16.9%)	0.609
Hypertension	236 (41.0%)	23 (29.9%)	0.064
Asthma	36 (6.3%)	4 (5.2%)	1.000
Inflammatory polyarthropathies	23 (4.0%)	6 (7.8%)	0.139
Concomitant medication			
Corticosteroids	154 (26.8%)	31 (40.2%)	0.021
Endocrine therapy	227 (39.5%)	41 (53.2%)	0.026
Immunostimulants	56 (9.7%)	14 (18.2%)	0.031
Lipid modifying agents	83 (14.4%)	15 (19.5%)	0.238
Proton pump inhibitors	233 (40.5%)	36 (46.8%)	0.325
VEGF	71 (12.3%)	18 (23.4%)	0.013
Total	574–576 (87.0%–89.5%)	76–78 (11.5%–13.0%)	

*P* value computed from Fisher’s exact test

*Due to secondary Use Act, frequency must be anonymous [24]

**Table 2:** Cox regression on factors associated with medication-related osteonecrosis of the jaw (MRONJ) in Finnish women with breast cancer

	Unadjusted model HR (95%)	Adjusted model AHR (95% CI)
Oral diseases		
Dental caries	2.19 (1.33–3.60) *	0.93 (0.51–1.71)
Pulpitis & periapical infection	2.87 (1.80–4.58) **	0.87 (0.46–1.65)
Periodontitis	6.76 (2.12–21.58) *	3.77 (1.06–13.37)*
Dental procedures		
Root canal treatment	6.07 (2.43–15.15) **	4.15 (1.39–12.41) *
Periodontal treatment	1.95 (0.78–4.83)	1.24 (0.47–3.31)
Tooth extraction	4.23 (2.7–6.61) **	3.99 (2.10–7.61) **
Antiresorptive drug		
Bisphosphonates	1	1
Denosumab	6.34 (1.54–26.01) *	6.40 (1.54–26.61) *
Both	4.17 (0.97–17.82)	3.75 (0.86–16.31)
Comorbidities		
Hypothyroidism	0.76 (0.35–1.66)	-
Diabetes	1.11 (0.61–2.02)	-
Hypertension	0.67 (0.41–1.08)	0.63 (0.38–1.06)
Asthma	0.97 (0.35–2.64)	-
Inflammatory polyarthropathies	2.00 (0.86–4.64)	-
Concomitant medication		
Corticosteroids	2.01 (1.27–3.20)**	2.12 (1.17–3.87)*
Endocrine therapy	1.50 (0.96–2.35)	1.05 (0.62–1.80)
Immunostimulants	1.61 (0.90–2.89)	1.09 (0.54–2.19)
Lipid modifying agents	1.27 (0.72–2.23)	-
Proton pump inhibitors	1.32 (0.84–2.07)	-
VEGFs	1.81 (1.07–3.07)*	0.96 (0.49–1.90)

Model 1 unadjusted model

Model 2 adjusted with age, oral diseases (dental caries, pulpitis & periapical infection, periodontitis), dental procedures (root canal treatment, periodontal treatment, tooth extraction), history of corticosteroids, endocrine therapy, history of immunostimulants and VEGF inhibitor therapy

Abbreviations: *HR (95% CI)* Hazard Ratio (95% confidence interval), *AHR (95% CI)* Adjusted hazard ratio (95% confidence interval), *Ref* reference group

*p-value < 0.05

** *p*-value < 0.001

**Table 3:** Incidence of medication-related osteonecrosis of the jaw (MRONJ) in women with breast cancer, according to antiresorptive treatment after 5–8 years follow-up period

Antiresorptive treatment	Number of patients	Patients without MRONJ	Patients with MRONJ	Incidence (%)
Bisphosphonates	85–89*	85–87	< 3*	0–2.2
Denosumab	427	373	54	12.6
Sequential use	137	116	21	15.3
Total	652	574–576*	76–78*	13.3

*Due to secondary Use Act, frequency must be anonymous [24]

In the unadjusted models, dental caries (HR 2.19, 95% CI: 1.33–3.60), pulpitis and/or periapical infection (HR 2.87, 95% CI: 1.80–4.58), and periodontitis (HR 6.76, 95% CI: 2.12–21.58) were associated with MRONJ. After adjusting for covariates, the HR values became lower. Root canal treatment (HR 6.07, 95% CI: 2.43–15.15; AHR 4.15, 95% CI: 1.39–12.41) and tooth extraction (HR 4.23, 95% CI: 2.70–6.61; AHR 3.99, 95% CI: 2.10–7.61) showed the strongest associations with MRONJ among the dental procedures examined. Concomitant corticosteroid use was also associated with MRONJ (HR 2.01, 95% CI: 1.27–3.20; AHR 2.12, 95% CI: 1.17–3.87) (Table 2).

Despite not being statistically significant, patients with existing comorbidities such as diabetes (HR 1.11, 95% CI: 0.61–2.02, *p* = 0.598) and inflammatory polyarthropathies (HR 2.00, 95% CI: 0.86–4.64, *p* = 0.130) might slightly elevate the risk of developing MRONJ. Endocrine therapy (AHR 1.05, 95% CI: 0.62 − 1.80, *p* = 0.838), immunostimulant use (AHR 1.09, 95% CI: 0.54–2.19, *p* = 0.817), lipid modifying agents use (HR 1.27, 95% CI: 0.72–2.23, *p* = 0.245), and proton pump inhibitors use (HR 1.32, 95% CI: 0.84–2.07, *p* = 0.297) were associated with modestly increased HR estimates, although none of these associations were statistically significant.

## Discussion

This study evaluated the incidence and associated factors of MRONJ among Finnish breast cancer patients with bone metastases receiving antiresorptive therapy between 2013 and 2015, with follow-up continuing until 2020. Based on the clinical context, patients were likely exposed to oncology-dose antiresorptive therapy rather than low-dose therapy. Exact dosing data were unavailable due to the nature of the study. The total incidence of MRONJ was 13.3%. The incidence ranged from 0% to 2.2% among bisphosphonate users, compared with 12.6% among denosumab users, and 15.3% among patients receiving sequential bisphosphonate and denosumab therapy. Denosumab use and tooth extraction showed the strongest associations with MRONJ. In addition, oral diseases and infection-related dental procedures were associated with MRONJ. However, tooth extraction likely reflects underlying oral infection and local inflammation rather than an independent causal mechanism. Furthermore, differences in treatment duration, cumulative exposure, and imbalance in treatment group sizes may have influenced the observed risk estimates.

Patients receiving antiresorptive therapy are often divided into two groups depending on the dosage: (a) high-dose patients with bone metastasis or multiple myeloma, and (b) low-dose patients with bone metabolic disease, such as osteoporosis. Patients in group (a) receive higher doses more frequently than group (b) and are, thus, more likely to develop MRONJ [17, 26]. Breast cancer patients may belong to both groups, since even without bone metastasis they often receive osteometabolic medications to prevent Cancer Treatment-Induced Bone Loss (CTIBL). CTIBL is caused by treatments such as gonadotropin-releasing hormone analogues, chemotherapy, and/or aromatase inhibitors, which lower estrogen levels, induce early menopause, and increase the risk of bone loss [27–29]. Our cohort likely consisted mainly of patients receiving oncology-dose antiresorptive therapy, as only patients with breast cancer and bone metastases were included, while patients with a concomitant osteoporosis diagnosis were excluded. However, exact dosing information was unavailable.

The total incidence of MRONJ in this study was higher than in most previous studies, with the exception of Brunner et al. [14, 16, 30–32]. The study by Hallmer et al. included a greater proportion of bisphosphonate users, which likely explains the lower overall incidence in that population [16]. In our analysis, the incidence among bisphosphonate users was low (0–2.2%) and was in line with previous studies by Brunner, Bamias, Hallmer, and Ikesue (2.8%, 2.9%, 4.1%, and 4.4% respectively) ^14–16,33^. The incidence among denosumab users (12.6%) and sequential users (15.3%) were consistent with other international findings [14, 15, 33]. Denosumab use was associated with a higher risk of MRONJ compared with bisphosphonate therapy (AHR 6.41, 95% CI: 1.54–26.61), although the estimate was imprecise. Sequential bisphosphonate–denosumab therapy showed a higher hazard ratio (AHR 3.75, 95% CI: 0.86–16.31), but the association was not statistically significant.

The role of dental infections and procedures on the development of MRONJ has been debated in earlier literature. Bassan et al. suggested that an inflammatory stimulus is necessary for MRONJ lesions to occur, while Brachhi et al. reported a fourfold risk increase when dental procedures were performed and a 23-fold risk increase when at least one tooth was extracted [12, 21]. Our findings support this view. Strong associations with MRONJ were observed for both tooth extraction and root canal treatment in the adjusted and unadjusted models. Periodontal treatment, on the other hand, was not significantly associated with MRONJ in this cohort. The observed associations between oral diseases and MRONJ are consistent with the hypothesis that local inflammation and impaired bone remodeling contribute to MRONJ pathogenesis. The results also support international recommendations emphasizing preventive dental care and multidisciplinary collaboration between oncologists and dental professionals before initiating antiresorptive therapy.

Apart from corticocosteroid use and VEGF inhibitor therapy, comorbidities and concomitant medications were not significantly associated with an increased risk of MRONJ. Compared with oral diseases and antiresorptive drug choice, these factors to play a less prominent role in MRONJ development. Although simultaneous use of certain drugs (e.g., proton pump inhibitors, lipid-modifying agents, immunostimulants, endocrine therapies) was associated with a slightly higher incidence, these associations were modest and secondary compared to oral health-related factors.

To our knowledge, the current study is the first population-based cohort study to specifically investigate breast cancer patients treated with antiresorptives in Finland. Accurate and large nationwide registries available in Finland enable comprehensive real-world studies. The strengths of this study include its retrospective, population-based cohort design, long follow-up period, and the inclusion of both general health and oral health covariates, providing a comprehensive view of MRONJ risks.

One limitation of this study was that some dental procedures were recorded for only a small number of patients, which reduced statistical power and limited detailed reporting due to the requirements of the Finnish Act on the Secondary Use of Health and Social Data [24]. In addition, this was a registry-based study, and therefore more detailed clinical information regarding MRONJ lesions and oral diseases was unavailable. MRONJ diagnoses were based on ICD-10 coding without individual clinical validation, which may have introduced some degree of misclassification. Furthermore, the ICD-10 code M87.1 may also include rare cases of medication-related osteonecrosis occurring outside the jaws, such as medication-related external auditory canal osteonecrosis [34]. However, this is unlikely to have substantially affected the results, as only a very small number of such cases have been reported globally in the literature. Although ICD-10 code C50 for breast cancer is widely and consistently used in Finnish healthcare, coding for bone metastases (C79.5) may be less comprehensive, potentially leading to underidentification of eligible patients. Because the temporal relationship between dental infections, dental procedures, and MRONJ onset could not be confirmed from registry data, causal interpretations of these associations should be made cautiously. Death as a competing event was not accounted for and may have influenced MRONJ risk estimates in this metastatic cancer population. Finally, detailed information regarding antiresorptive treatment dose, duration, cumulative exposure, and administration intervals was not available, limiting further evaluation of treatment-related MRONJ risk. The small number of MRONJ events among bisphosphonate users should also be considered when interpreting the differences between antiresorptive treatment groups. Although higher HR estimates were observed for denosumab and sequential therapy compared with bisphosphonate therapy, the low number of events in the bisphosphonate reference group resulted in wide confidence intervals and reduced precision of these estimates. These findings should therefore be interpreted cautiously, particularly regarding the magnitude of the differences between treatment groups. In addition, some established systemic risk factors for MRONJ, including smoking, renal impairment, and chemotherapy exposure, were not available or could not be reliably identified from the registry data. Therefore, residual confounding may have influenced the observed associations, particularly regarding systemic risk factors and concomitant medications.Few studies have included equally long follow-up times, even though the onset of MRONJ may occur over 40 months after initiation of antiresorptive treatment [17, 35]. The risk is known to increase with each year of continued medication use. In further studies, it would be useful to record more detailed information on patients’ oral hygiene practices and to extend the follow-up period, which could also reduce the current imbalance in case numbers between denosumab and bisphosphonate-treated patients. However, if the onset of MRONJ occurs mostly after five years, the clinical impact may be limited in the breast cancer patients, who are on average over 70 years old and face an 18% ten-year mortality [36]. We recommend that future studies should also examine the influence of preventive oral care on the onset of MRONJ. As a clinical consideration, we emphasize the importance of continuing indicated antiresorptive therapy despite the risk of MRONJ. Discontinuation of denosumab may lead to rapid bone loss and a rebound increase in bone turnover, with an increased risk of vertebral fractures. Therefore, decisions regarding the continuation or discontinuation of antiresorptive therapy should be carefully considered on an individual basis, balancing the potential risk of MRONJ against the benefits of treatment and the risks associated with its discontinuation.

### Clinical implications

For dental clinicians, this study underlines the importance of early detection and treatment of dental infections before and during the administration of AR medication and good oral hygiene to reduce the risk of MRONJ. For general health practitioners, this study underscores the need to weigh the benefits and risks of each antiresorptive therapy and consulting a dental professional who is familiar with MRONJ before the administration.

## Conclusions

In conclusion, this registry-based study with pseudonymized data provides novel population-level evidence on the incidence of MRONJ and its associated factors among antiresorptive-treated breast cancer patients in Finland. Our findings are in line with international evidence and highlight a strong association between denosumab use and MRONJ. Oral infections, oral diseases, and related dental procedures were also associated with MRONJ and should be considered in clinical decision-making. Despite these findings, antiresorptive therapies remain an important part of the management of metastatic breast cancer.

## Data Availability

The data that support the findings of this study can be requested from the Finnish Social and Health Data Permit Authority (Findata; THL/3317/14.02.00/2021).

## Declarations

### Prior presentation

Accepted for poster presentation at the IADR General Session, March 25, 2026 (San Diego, CA).

## References

[1] World Health Organization (2024) Breast cancer. https://www.who.int/news-room/fact-sheets/detail/breast-cancer. Accessed 5 Jul 2025

[2] Sung H, Ferlay J, Siegel RL et al (2021) Global Cancer Statistics 2020: GLOBOCAN Estimates of Incidence and Mortality Worldwide for 36 Cancers in 185 Countries. CA Cancer J Clin 71:209–249. 10.3322/caac.21660 33538338

[3] Finnish Cancer Registry (2022) Finnish Cancer Registry 2022. https://syoparekisteri.fi/assets/themes/ssy3/factsheets/cancer_in_finland_2022.html. Accessed 5 Jul 2025

[4] Hiraga T, Williams PJ, Ueda A et al (2004) Zoledronic Acid Inhibits Visceral Metastases in the 4T1/luc Mouse Breast Cancer Model. Clin Cancer Res 10:4559–4567. 10.1158/1078-0432.CCR-03-0325 15240548

[5] Moshi MR, Nicolopoulos K, Stringer D et al (2023) The Clinical Effectiveness of Denosumab (Prolia^®^) for the Treatment of Osteoporosis in Postmenopausal Women, Compared to Bisphosphonates, Selective Estrogen Receptor Modulators (SERM), and Placebo: A Systematic Review and Network Meta-Analysis. Calcif Tissue Int 112:631–646. 10.1007/s00223-023-01078-z 37016189

[6] Eisen A, Somerfield MR, Accordino MK et al (2022) Use of Adjuvant Bisphosphonates and Other Bone-Modifying Agents in Breast Cancer: ASCO-OH (CCO) Guideline Update. J Clin Oncol 40:787–800. 10.1200/JCO.21.02647 35041467

[7] Ruggiero SL, Dodson TB, Fantasia J et al (2014) American Association of Oral and Maxillofacial Surgeons Position Paper on Medication-Related Osteonecrosis of the Jaw—2014 Update. J Oral Maxillofac Surg 72:1938–1956. 10.1016/j.joms.2014.04.031 25234529

[8] Ruggiero SL, Dodson TB, Aghaloo T et al (2022) American Association of Oral and Maxillofacial Surgeons’ Position Paper on Medication-Related Osteonecrosis of the Jaws—2022 Update. J Oral Maxillofac Surg 80:920–943. 10.1016/j.joms.2022.02.008 35300956

[9] do N Poubel VL, Silva CAB, Mezzomo LAM et al (2018) The risk of osteonecrosis on alveolar healing after tooth extraction and systemic administration of antiresorptive drugs in rodents: a systematic review. J Cranio-Maxillofacial Surg 46:245–256. 10.1016/j.jcms.2017.11.008 29233703

[10] Rytkonen E, Ottavainen V, Rytkönen A et al (2018) Denosumab treatment for aggressive multiple recurrent familial central giant-cell granulomas. Ann Maxillofac Surg 8:265. 10.4103/ams.ams_192_18 PMC6327812 30693243

[11] Patel V, Mansi J, Ghosh S et al (2018) MRONJ risk of adjuvant bisphosphonates in early stage breast cancer. Br Dent J 224:74–79. 10.1038/sj.bdj.2017.1039 29242516

[12] Bracchi P, Zecca E, Brunelli C et al (2023) A real-world study on the prevalence and risk factors of medication related osteonecrosis of the jaw in cancer patients with bone metastases treated with Denosumab. Cancer Med 12:18317–18326. 10.1002/cam4.6429 PMC10523953 37559413

[13] Limones A, Sáez-Alcaide LM, Díaz-Parreño SA et al (2020) Medication-related osteonecrosis of the jaws (MRONJ) in cancer patients treated with denosumab VS. zoledronic acid: a systematic review and meta-analysis. Med Oral Patol Oral Cir Bucal e326–e336. 10.4317/medoral.23324 PMC7211372 32271321

[14] Brunner C, Arvandi M, Marth C et al (2025) Incidence of Medication-Related Osteonecrosis of the Jaw in Patients With Breast Cancer During a 20-Year Follow-Up: A Population-Based Multicenter Retrospective Study. J Clin Oncol 43:180–188. 10.1200/JCO.24.00171 PMC11708989 39163561

[15] Ikesue H, Doi K, Morimoto M et al (2021) Switching from zoledronic acid to denosumab increases the risk for developing medication-related osteonecrosis of the jaw in patients with bone metastases. Cancer Chemother Pharmacol 87:871–877. 10.1007/s00280-021-04262-w PMC8110486 33791853

[16] Hallmer F, Bjarnadottir O, Götrick B et al (2020) Incidence of and risk factors for medication-related osteonecrosis of the jaw in women with breast cancer with bone metastasis: a population-based study. Oral Surg Oral Med Oral Pathol Oral Radiol 130:252–257. 10.1016/j.oooo.2020.04.808 32536575

[17] Kujanpää M, Vuollo V, Tiisanoja A et al (2025) Incidence of medication-related osteonecrosis of the jaw and associated antiresorptive drugs in adult Finnish population. Sci Rep 15:17377. 10.1038/s41598-025-02225-2 PMC12089364 40389539

[18] McGowan K, McGowan T, Ivanovski S (2018) Risk factors for medication-related osteonecrosis of the jaws: A systematic review. Oral Dis 24:527–536. 10.1111/odi.12708 28656643

[19] Khan AA, Morrison A, Hanley DA et al (2015) Diagnosis and Management of Osteonecrosis of the Jaw: A Systematic Review and International Consensus. J Bone Miner Res 30:3–23. 10.1002/jbmr.2405 25414052

[20] Thumbigere-Math V, Tu L, Huckabay S et al (2012) A Retrospective Study Evaluating Frequency and Risk Factors of Osteonecrosis of the Jaw in 576 Cancer Patients Receiving Intravenous Bisphosphonates. Am J Clin Oncol 35:386–392. 10.1097/COC.0b013e3182155fcb 22561331

[21] Bassan Marinho Maciel G, Marinho Maciel R, Linhares Ferrazzo K, Cademartori Danesi C (2024) Etiopathogenesis of medication-related osteonecrosis of the jaws: a review. J Mol Med 102:353–364. 10.1007/s00109-024-02425-9 38302741

[22] Khamaisi M, Regev E, Yarom N et al (2007) Possible Association between Diabetes and Bisphosphonate-Related Jaw Osteonecrosis. J Clin Endocrinol Metab 92:1172–1175. 10.1210/jc.2006-2036 17179196

[23] Nicoletti P, Cartsos VM, Palaska PK et al (2012) Genomewide Pharmacogenetics of Bisphosphonate-Induced Osteonecrosis of the Jaw: The Role of *RBMS3*. Oncologist 17:279–287. 10.1634/theoncologist.2011-0202 PMC3286178 22267851

[24] Finlex (2019) Laki sosiaali- ja terveystietojen toissijaisesta käytöstä. In: Laki sosiaali- ja terveystietojen toissijaisesta käytöstä. https://www.finlex.fi/en/legislation/2019/552#Text%20of%20statute. Accessed 26 Mar 2024

[25] World Health Organization (n.d.b) Anatomical Therapeutic Chemical (ATC) Classification

[26] Hallmer F, Andersson G, Götrick B et al (2018) Prevalence, initiating factor, and treatment outcome of medication-related osteonecrosis of the jaw—a 4-year prospective study. Oral Surg Oral Med Oral Pathol Oral Radiol 126:477–485. 10.1016/j.oooo.2018.08.015 30249535

[27] Handforth C, D’Oronzo S, Coleman R, Brown J (2018) Cancer Treatment and Bone Health. Calcif Tissue Int 102:251–264. 10.1007/s00223-017-0369-x PMC5805796 29353450

[28] Coleman R, Hadji P, Body J-J et al (2020) Bone health in cancer: ESMO Clinical Practice Guidelines. Ann Oncol 31:1650–1663. 10.1016/j.annonc.2020.07.019 32801018

[29] Diana A, Carlino F, Giunta EF et al (2021) Cancer Treatment–Induced Bone Loss (CTIBL): State of the Art and Proper Management in Breast Cancer Patients on Endocrine Therapy. Curr Treat Options Oncol 22:45. 10.1007/s11864-021-00835-2 PMC8052225 33864145

[30] Coleman R, Finkelstein DM, Barrios C et al (2020) Adjuvant denosumab in early breast cancer (D-CARE): an international, multicentre, randomised, controlled, phase 3 trial. Lancet Oncol 21:60–72. 10.1016/S1470-2045(19)30687-4 31806543

[31] Coleman R, Cameron D, Dodwell D et al (2014) Adjuvant zoledronic acid in patients with early breast cancer: final efficacy analysis of the AZURE (BIG 01/04) randomised open-label phase 3 trial. Lancet Oncol 15:997–1006. 10.1016/S1470-2045(14)70302-X 25035292

[32] Valachis A, Polyzos NP, Coleman RE et al (2013) Adjuvant Therapy With Zoledronic Acid in Patients With Breast Cancer: A Systematic Review and Meta-Analysis. Oncologist 18:353–361. 10.1634/theoncologist.2012-0261 PMC3639520 23404816

[33] Ikesue H, Doi K, Morimoto M et al (2022) Risk evaluation of denosumab and zoledronic acid for medication-related osteonecrosis of the jaw in patients with bone metastases: a propensity score–matched analysis. Support Care Cancer 30:2341–2348. 10.1007/s00520-021-06634-7 PMC8794983 34738163

[34] Khan I (2024) Medication-related osteonecrosis of the external auditory canal – A rapid review of the literature and relevance to special care dentists. Spec Care Dentist 44:314–327. 10.1111/scd.12893 37384433

[35] Fusco V, Cabras M, Erovigni F et al (2021) A multicenter observational study on Medication-Related Osteonecrosis of the Jaw (MRONJ) in advanced cancer and myeloma patients of a cancer network in North-Western Italy. Med Oral Patol Oral Cir Bucal e466–e473. 10.4317/medoral.24318 PMC8254890 33340073

[36] Early Breast Cancer Trialists’ Collaborative Group (2015) Adjuvant bisphosphonate treatment in early breast cancer: meta-analyses of individual patient data from randomised trials. Lancet 386:1353–1361. 10.1016/S0140-6736(15)60908-4 26211824

